# Nutritional, sleep, physical activity, and quality-of-life changes during Ramadan fasting: a prospective comparative study

**DOI:** 10.3389/fnut.2026.1809040

**Published:** 2026-05-04

**Authors:** Eftal Geçgil Demir, Canel Öner Sayar, Rabia Melda Karaağaç, Nihal Zekiye Erdem, Nadide Gizem Tarakçı Filiz, Emre Bayraktaroğlu, Rüken Aslınur Samancı, Elif Mızrak

**Affiliations:** 1 Department of Nutrition and Dietetics, School of Health Sciences, Istanbul Medipol University, Istanbul, Türkiye; 2 Department of Nutrition and Dietetics, Faculty of Health Sciences, Fenerbahçe University, Istanbul, Türkiye

**Keywords:** nutrition, physical activity, quality of life, Ramadan, sleep

## Abstract

**Background:**

Ramadan is the holiest month in the Islamic calendar. Ramadan fasting, which lasts from sunrise to sunset, significantly affects eating habits, physical activity, daytime sleepiness, and quality of life. In this study, body weight, daytime sleepiness (Epworth Sleepiness Scale), physical activity level, dietary intake, and quality of life (SF-36) were assessed in fasting and non-fasting individuals before, during, and after Ramadan.

**Methods:**

In this prospective longitudinal comparative study of 282 healthy adults, individuals were divided into fasting and non-fasting groups. Data were collected in four phases: 1 week before, in the middle, at the end, and 2 weeks after Ramadan.

**Results:**

Total energy intake, macronutrient consumption, and body weight decreased during Ramadan and increased again after Ramadan, with these changes being more pronounced in the fasting group. Daytime sleepiness increased and physical activity level decreased in fasting individuals during Ramadan, but both returned toward baseline levels after Ramadan. In terms of quality of life, significant differences between fasting and non-fasting individuals were observed, particularly in physical and emotional role functioning domains (*p* < 0.05).

**Conclusion:**

This study demonstrated that Ramadan fasting is associated with changes in dietary intake, daytime sleepiness, physical activity, and quality of life. Moreover, similar patterns observed in non-fasting individuals suggest that environmental and lifestyle factors during Ramadan may also play a role.

## 1 Introduction

Ramadan is the holiest month in the Islamic calendar (1). During Ramadan fasting, Muslims are prohibited from eating, drinking anything, including water, taking medication, and engaging in sexual activity during certain hours from morning to evening. During Ramadan, individuals abstain from food and drink from dawn to dusk. The pre-dawn meal is called suhoor, whereas the meal consumed after sunset to break the fast is referred to as iftar (2–4). The Muslim population is expected to grow to 2.2 billion by 2030, representing 26.4% of the world’s estimated total population. Although there are other types of fasting in Islam, Ramadan fasting is obligatory for every Muslim if they meet certain criteria (5). From puberty onwards, healthy male and female Muslims are required to fast. In Ramadan, the ninth month of the Islamic calendar, fasting is observed for approximately 29 to 30 days per year, with daily fasting periods ranging from 10 to 19 h, reaching 21 h depending on geographical location and solar season (6). During the fasting period, there are significant changes in dietary habits and food consumption patterns, as well as changes in physical activity levels, daytime sleepiness, and quality of life (1, 4, 5, 7). Food intake during Ramadan is an issue that needs to be specifically analyzed in terms of the timing and frequency of meals. Food intake during Ramadan has been associated with major changes in dietary patterns, food groups, energy, and macro- and micronutrient intakes (8). For example, Shatila et al. found that average energy intake was slightly higher during Ramadan, while the proportions of total daily energy intake from macronutrients were similar (protein ~15%, carbohydrate ~45%, and fat ~40%). Some evidence suggests that Ramadan fasting has a beneficial effect on body weight (9). Jahrami et al. found that participants who fasted during Ramadan had a significant reduction in body weight, with a decrease of 1.87 kg, body mass index (BMI) of 0.69 kg/m^2^, and fat mass of 0.87% (9). It is also thought that increased spiritual activity during this month may have a positive effect on emotional and mental health (4). A detailed investigation of the differences in physical activity, daytime sleepiness, and food consumption of Muslim individuals during this period is necessary to understand the effects of Ramadan fasting (10). Although previous studies have examined the effects of Ramadan fasting on dietary intake, physical activity, and sleep-related outcomes, most have focused only on fasting individuals and have often used cross-sectional or limited follow-up designs. Moreover, comparative longitudinal data including both fasting and non-fasting individuals across multiple time points before, during, and after Ramadan remain limited. Therefore, this study aimed to examine the body weight, daytime sleepiness, quality of life, dietary habits, and physical activity levels of fasting and non-fasting individuals before, during, and after Ramadan.

## 2 Materials and methods

### 2.1 Sample selection and procedure

This study was a prospective longitudinal comparative study designed to include two groups: individuals aged 20–55 years, living in Istanbul, healthy (without any disease and not taking any medication, not following any special diet, for female participants; not pregnant or lactating), intending to fast for the whole month of Ramadan and having fasted at least once before, and non-fasting individuals meeting the same criteria. Participants were recruited in Istanbul through a face-to-face approach by the research team. Individuals who met the eligibility criteria and voluntarily agreed to participate were enrolled in the study after providing written informed consent. According to the 90% power analysis, the minimum sample size was set at 240, with 120 people from each group. In the sample size calculation, the type I error (*α*) was 0.05 and the effect size was 0.15 (11). Power analysis was performed using G-Power (ver. 3.1.9.4). To account for attrition, a target of 20% more than the number obtained was set, and the study was conducted with a total of 282 people. The final analytic sample comprised 282 participants. Before enrollment, all participants were informed about the study in a face-to-face interview, and written informed consent was obtained using the voluntary information and consent form. The study was approved by the Istanbul Medipol University Non-Interventional Clinical Research Ethics Committee, E-10840098-202.3.02-5321, with decision number 803. Measurements in the study were taken 1 week before (T1), in the middle (T2), at the end (T3), and 2 weeks after the end of Ramadan (T4). Daytime sleepiness, physical activity, dietary intake, and body weight were assessed at four time points: 1 week before Ramadan, mid-Ramadan, end of Ramadan, and 2 weeks after Ramadan. Quality of life was assessed at three time points: 1 week before Ramadan, mid-Ramadan, and 2 weeks after Ramadan. Due to the length and comprehensive nature of the quality of life questionnaire, it was administered at three time points to reduce participant burden, while still allowing the evaluation of temporal changes. As fasting duration varies depending on seasonal and geographical factors, Ramadan in 2025 in Istanbul (March 1–March 29) corresponded to a daylight duration ranging approximately from 11.4 to 12.9 h, with an average of about 12.2 h.

Data collection was conducted through face-to-face interviews by trained researchers. All assessments at each data collection point were conducted face-to-face. Blinding of the interviewers to the study group was not feasible because fasting status was inherent to the study design and directly relevant to the interviews. Data were recorded using structured study forms and subsequently entered into an electronic database by the research team. Participant information was coded and stored in password-protected files accessible only to the researchers to ensure confidentiality and data security. Completing all questionnaires required approximately 20–25 min per participant. Questionnaires were checked for completeness immediately after collection to minimize missing data. When incomplete information was identified, participants were contacted by phone to obtain the missing data and ensure dataset completeness.

### 2.2 Data collection tools

Questionnaire on demographic information and anthropometric measurements: Demographic information, including sex, age, smoking, and alcohol consumption, marital, and educational status of the individuals were asked. Height (cm) and body weight (kg) were measured by trained researchers at each time point in a standardized clinical setting at our research center. Body weight was measured using a calibrated digital scale, with participants wearing light clothing and no shoes. Height was measured using a stadiometer, with participants standing upright in the Frankfurt plane. BMI was calculated as weight (kg) divided by height squared (m^2^). Participants were instructed to attend measurements in a fasting state and, where possible, after voiding.

Epworth Sleepiness Scale (ESS): This was used to assess the sleep status of the participants. The scale was developed by M. W. Johns in 1991 and consists of eight questions (12). It is scored as 0, 1, 2, and 3, and a score of 11 and above indicates excessive daytime sleepiness. It has been found that the ESS is a valid and reliable scale for assessing the general level of sleepiness, and it is a valid and reliable test with high internal consistency (Cronbach’s alpha = 0.80) for eight different conditions that can be used in studies on sleep and sleep disorders in Turkey (13).

Physical Activity Assessment: The type and duration of physical activity of all participants were assessed using a 24-h recall method. The energy expenditure for each activity was calculated by multiplying the activity-specific physical activity rate, the duration of the activity (minutes), and the basal metabolic rate per hour. Basal metabolic rate was calculated using the Schofield equation. Total daily energy expenditure was obtained by summing the energy expenditure of all reported activities. Physical activity level (PAL) was calculated by dividing total daily energy expenditure by basal metabolic rate. PAL values were classified as sedentary or less active (1.4–1.5), moderately active (1.6–1.7), active (1.8–1.9), and very active (≥2.0), according to the Turkey Nutrition Guide (14).

Quality of Life Scale (SF-36): Participants’ quality of life was assessed using the SF-36. Developed by Ware and colleagues, the SF-36 aims to assess quality of life with multidimentions (15). The Turkish validity and reliability study of the scale was conducted by Koçyiğit et al. and adapted to the Turkish language (16). The scale consists of eight subdimensions: physical function, social function, physical role, emotional role, mental health, energy/vitality, pain, and general health perception. The subdimensions were scored between 0 and 100 points. The higher the total score, the better the quality of life. The Cronbach’s alpha value of the scale for this study was found to be 0.91. The data collection forms used in the study, including the dietary recall form, a 24-h recall physical activity, and the SF-36 scale, are provided in the Supplementary Data Sheet 1–4.

### 2.3 Food record

Participants were provided with a pre-designed food recall form to assess their dietary intake. During the first interview, the researcher explained how to complete the food recall form and provided portion size estimation training to improve the accuracy of the dietary records. Participants were instructed on how to estimate portion sizes using common household measures and visual examples. Participants were asked to complete a 24-h food record at four different time points. The dietary data obtained from the food records were analyzed using the BeBiS 9 Nutrition Information System (BeBiS, Istanbul, Turkey). Daily energy intake and macronutrient composition, including carbohydrates, proteins, and fats, were calculated in grams and as percentages of total energy intake.

### 2.4 Statistical analysis

Data analysis was performed using the SPSS 22.0 software package. The distribution of continuous variables was assessed using the Kolmogorov–Smirnov and Shapiro–Wilk tests; furthermore, skewness and kurtosis values falling within the range of ±1.5 were accepted as an indicator of normal distribution. Continuous variables are presented as mean and standard deviation; categorical variables are presented as counts and percentages. For initial comparisons between groups, the Chi-square test was used for categorical variables; where expected cell frequencies were insufficient, Fisher’s Exact test was applied, and for multi-cell tables, the Fisher–Freeman–Halton exact test was used. For comparisons of continuous variables between two independent groups, the independent samples *t*-test was used. To assess longitudinal changes, a two-way repeated measures analysis of variance (ANOVA) was used, with time (T1: pre-Ramadan, T2: mid-Ramadan, T3: end of Ramadan, T4: 2 weeks post-Ramadan) as the within-subjects factor and group (fasting/non-fasting) as the between-subjects factor. The analyses examined the effects of time, group, and the time × group interaction. To control for the effects of potential confounding variables, age and pre-Ramadan body weight were included in the model as covariates (repeated measures ANCOVA approach). The sphericity assumption was assessed using the Mauchly test, and the Greenhouse–Geisser correction was applied where the assumption was violated. Where a significant main effect or interaction was detected, multiple comparisons were performed using the Bonferroni correction, and differences between time points within groups were indicated using appropriate superscripts. To enhance clinical interpretability, change (*Δ*) values relative to baseline were calculated for all outcome variables. In this context, change scores were obtained for the T2–T1, T3–T1, and T4–T1 time intervals; for the SF-36 subscales measured at the three time points, comparisons were made between T2–T1 and T4–T1. An independent samples *t*-test was used for between-group comparisons of change scores. Results were reported alongside the mean difference and 95% confidence interval. In all analyses, a two-tailed *p*-value of <0.05 was considered statistically significant.

## 3 Results

Table 1 presents the distribution of participants’ descriptive characteristics according to fasting status. The mean age of the fasting group was significantly higher than that of the non-fasting group (32.71 ± 7.10 vs. 30.92 ± 6.53 years, *p* = 0.028). Smoking status differed significantly between the groups, with a higher proportion of smokers in the non-fasting group (*p* = 0.001). Alcohol consumption was also significantly higher in the non-fasting group than the fasting group (*p* < 0.001). Marital status showed a significant difference between the groups, with a higher proportion of married individuals in the fasting group (*p* = 0.002).

**Table 1:** Distribution of descriptive characteristics and comparisons by group.

Variables	Groups		*p
	Fasting (*n* = 141) Mean ± SD	Non fasting (*n* = 141) Mean ± SD	
Age	32.71 ± 7.1	30.92 ± 6.53	**0.028**
Height	171.95 ± 10.55	172.34 ± 9.67	0.747
	*n* (%)	*n* (%)	**p
Sex			
Female	72 (51.1%)	70 (49.6%)	***0.453
Male	69 (48.9%)	71 (50.4%)	
Smoking status			
Yes	45 (31.9%)	71 (50.4%)	***0.001
No	96 (68.1%)	70 (49.6%)	
Alcohol status			
Yes	24 (17%)	82 (58.2%)	*** < 0.001
No	117 (83%)	59 (41.8%)	
Marital status			
Married	78 (55.3%)	53 (37.6%)	***0.002
Single	63 (44.7%)	88 (62.4%)	
Education status			
Literate	–	1 (0.7%)	****0.058
Primary education	12 (8.5%)	4 (2.8%)	
High school	38 (27%)	29 (20.6%)	
License	91 (64.5%)	107 (75.9%)	

* Student *t* test, ** Chi-Square test, *** Fisher’s Exact test, **** Fisher–Freeman–Halton exact test. Bold values indicate statistically significant differences (*p* < 0.05).

**Figure 1:**
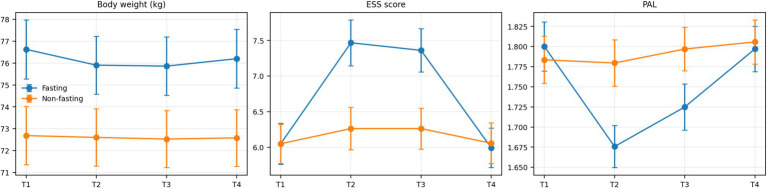
Temporal changes in body weight, daytime sleepiness (ESS), and physical activity levels in fasting and non-fasting individuals across four time points (T1: pre-Ramadan, T2: mid-Ramadan, T3: end-Ramadan, T4: 2 weeks post-Ramadan). Values are presented as mean ± standard error.

Changes in body weight, daytime sleepiness (ESS), and physical activity during the study period are presented in Table 2. The fact that the time × group interaction was significant for all these variables (*p* ≤ 0.004) indicates that the patterns of change over time differed between the fasting and non-fasting groups. In the fasting group, body weight decreased gradually throughout Ramadan, reaching its lowest levels in the middle and at the end of the month (ΔT2–T1: −0.72 kg; ΔT3–T1: −0.76 kg). Following Ramadan, a rise in body weight was observed, although it remained slightly below the baseline level. In contrast, body weight in the non-fasting group remained largely stable over time. In comparisons between the groups, it was found that weight loss during Ramadan was significantly greater in the fasting group, but that this difference disappeared after Ramadan. A different pattern of change was observed with regard to daytime sleepiness. In the fasting group, ESS scores increased throughout Ramadan (ΔT2–T1: +1.42; ΔT3–T1: +1.31) and returned to baseline levels after Ramadan. In the non-fasting group, however, only limited changes in ESS scores were observed. These findings indicate that the increase in daytime sleepiness during the Ramadan period is more pronounced in individuals who fast. A different pattern of change was observed with regard to daytime sleepiness. In the fasting group, ESS scores increased throughout Ramadan (ΔT2–T1: +1.42; ΔT3–T1: +1.31) and returned to baseline levels after Ramadan. In the non-fasting group, however, only limited changes in ESS scores were observed. These findings indicate that the increase in daytime sleepiness during the Ramadan period is more pronounced in individuals who fast. The temporal trajectories of body weight, daytime sleepiness, and physical activity across the four time points are illustrated in Figure 1.

**Table 2:** Comparison of body weight, ESS, and physical activity within and between groups.

Time point	Fasting (*n* = 141) Mean ± SD	Non-fasting (*n* = 141) Mean ± SD	p*	Time	Group	Time × Group
Body weight (kg)				**<0.001**	0.059	**0.004**
T1	76.63 ± 16.02ᵃ	72.69 ± 15.8ᵃ	**0.038**			
T2	75.91 ± 15.73ᵇ	72.60 ± 15.52ᵃ	0.076			
T3	75.86 ± 15.93ᵇ	72.53 ± 15.52ᵃ	0.076			
T4	76.20 ± 15.92ᵃ	72.58 ± 15.4ᵃ	0.053			
ΔT2–T1 (95% CI)	−0.72 (−0.99 to −0.46)	−0.08 (−0.29 to 0.12)				
ΔT3–T1 (95% CI)	−0.76 (−1.12 to −0.41)	−0.16 (−0.45 to 0.14)				
ΔT4–T1 (95% CI)	−0.43 (−0.77 to −0.08)	−0.10 (−0.45 to 0.24)				
Within-group comparison (p**)	**<0.001**	0.211				
ESS score				**<0.001**	0.134	**<0.001**
T1	6.05 ± 3.4ᵃ	6.05 ± 3.26ᵃ	1.000			
T2	7.47 ± 3.82ᵇ	6.26 ± 3.54ᵇ	**0.006**			
T3	7.36 ± 3.61ᵇ	6.26 ± 3.43ᵇ	**0.010**			
T4	5.99 ± 3.31ᵃ	6.06 ± 3.39ᵃ	0.865			
ΔT2–T1 (95% CI)	1.42 (0.92 to 1.91)	0.21 (−0.16 to 0.59)				
ΔT3–T1 (95% CI)	1.31 (0.85 to 1.77)	0.21 (−0.11 to 0.53)				
ΔT4–T1 (95% CI)	−0.06 (−0.46 to 0.35)	0.01 (−0.42 to 0.43)				
Within-group comparison (p**)	**<0.001**	**0.004**				
PAL				**<0.001**	0.239	**<0.001**
T1	1.80 ± 0.36ᵃ	1.78 ± 0.35ᵃ	0.692			
T2	1.68 ± 0.31ᵇ	1.78 ± 0.34ᵃ	**0.010**			
T3	1.72 ± 0.34ᵇ	1.80 ± 0.32ᵃ	**0.048**			
T4	1.80 ± 0.33ᵃ	1.81 ± 0.33ᵃ	0.873			
ΔT2–T1 (95% CI)	−0.12 (−0.17 to −0.08)	−0.00 (−0.04 to 0.03)				
ΔT3–T1 (95% CI)	−0.08 (−0.12 to −0.03)	0.01 (−0.03 to 0.06)				
ΔT4–T1 (95% CI)	−0.00 (−0.05 to 0.04)	0.02 (−0.02 to 0.06)				
Within-group comparison (p**)	**<0.001**	**0.032**				

Analyses were performed using two-way repeated measures ANOVA. Δ difference values represent the between-group difference in change from baseline (fasting minus non-fasting). Change scores (Δ) were calculated for each time interval (T2–T1, T3–T1, and T4–T1). Between-group comparisons of these change scores were performed using an independent samples *t*-test. In addition, repeated measures ANOVA was used to assess overall time, group, and time × group interaction effects. Values are presented as mean difference with 95% confidence intervals (CI). A *p*-value <0.05 was considered statistically significant. T1: pre-Ramadan; T2: mid-Ramadan; T3: end of Ramadan; T4: 2 weeks after Ramadan, a,b,c,d Post-hoc Bonferrini. a: There is a significant difference with T1. b: There is a significant difference with T2. c: There is a significant difference with T3. d: There is a significant difference with T4. Covariates: Age and pre-Ramadan body weight. Bold values indicate statistically significant differences (*p* < 0.05).

Comparisons of change scores between groups (Table 3) suggest that the observed interaction effects are largely attributable to more pronounced changes specific to the Ramadan period in the group that fasted.

**Table 3:** Between-group differences in change scores.

Variable	Comparison	Δ difference (Fasting – Non-fasting)	*p*
Body weight (kg)	ΔT2–T1	−0.64 (−0.97 to −0.30)	**<0.001**
	ΔT3–T1	−0.61 (−1.06 to −0.15)	**0.009**
	ΔT4–T1	−0.32 (−0.80 to 0.16)	0.193
ESS score	ΔT2–T1	1.21 (0.59 to 1.82)	**<0.001**
	ΔT3–T1	1.10 (0.54 to 1.66)	**<0.001**
	ΔT4–T1	−0.06 (−0.65 to 0.52)	0.829
PAL	ΔT2–T1	−0.12 (−0.18 to −0.06)	**<0.001**
	ΔT3–T1	−0.09 (−0.15 to −0.02)	**0.007**
	ΔT4–T1	−0.03 (−0.09 to 0.04)	0.423

Δ difference values represent the between-group difference in change from baseline (fasting minus non-fasting). Change scores (Δ) were calculated for each time interval (T2–T1, T3–T1, and T4–T1), and between-group comparisons were performed using an independent samples *t*-test. These analyses complement the repeated-measures framework by quantifying the magnitude and direction of change over time. Values are expressed as mean difference with 95% confidence intervals (CIs). Negative values indicate a greater reduction in the fasting group, and positive values indicate a greater increase. A two-sided *p*-value <0.05 was considered statistically significant. Bold values indicate statistically significant differences (*p* < 0.05).

Quality of life was assessed using the SF-36 subscales, and the results are presented in Table 4. A significant time × group interaction was observed in several subscales, indicating that there were different patterns of change between the two groups. Physical functioning levels decreased in the fasting group during Ramadan (ΔT2–T1: −4.94), but returned to levels close to baseline after Ramadan. In the non-fasting group, only minimal changes were observed. A more pronounced decrease was noted in the physical role difficulty subscale; this value fell significantly among individuals fasting during Ramadan (ΔT2–T1: −28.9) and subsequently recovered. Emotional distress decreased in the group that fasted, while the group that did not fast showed a slight upward trend. Similarly, social functioning declined in individuals who fasted during Ramadan, before subsequently improving. These findings suggest that certain aspects of quality of life are temporarily adversely affected during the fasting period. Although vitality scores decreased in both groups, this decrease was more pronounced in the fasting group and increased after Ramadan, indicating a significant interaction. In contrast, no significant changes over time or marked differences between the groups were observed in terms of mental health, pain, and perceived general health. The temporal changes in SF-36 subdimensions are also illustrated in Supplementary Figure S1.

**Table 4:** Comparison of groups by total SF-36 subdimension scores.

Time point	Fasting (*n* = 141) Mean ± SD	Non-fasting (*n* = 141) Mean ± SD	p*	Time	Group	Time × Group
Physical functioning						
T1	89.54 ± 14.19ᵃ	89.40 ± 13.73ᵃ	0.937	**<0.001**	0.539	**0.008**
T2	84.60 ± 17.69ᵇ	88.15 ± 17.91ᵃ	**0.041**			
T4	88.12 ± 17.41ᵃ	88.07 ± 17.91ᵃ	0.982			
ΔT2–T1 (95% CI)	−4.94 (−6.87 to −3.01)	−1.25 (−3.22 to 0.71)				
ΔT4–T1 (95% CI)	−1.42 (−3.09 to 0.25)	−1.33 (−3.54 to 0.88)				
Within-group comparison (p**)	**<0.001**	0.214				
Physical role functioning						
T1	80.67 ± 29.94ᵃ	77.41 ± 31.54ᵃ	0.354	**<0.001**	0.096	**<0.001**
T2	51.77 ± 40.49ᵇ	76.95 ± 34.34ᵃ	**<0.001**			
T4	78.55 ± 33.08ᵃ	73.49 ± 34.83ᵃ	0.192			
ΔT2–T1 (95% CI)	−28.90 (−36.03 to −21.77)	−0.46 (−5.10 to 4.18)				
ΔT4–T1 (95% CI)	−2.13 (−6.91 to 2.66)	−3.92 (−7.66 to −0.18)				
Within-group comparison (p**)	**<0.001**	**0.041**				
Emotional role functioning						
T1	76.24 ± 35.05ᵃ	69.25 ± 38.44ᵃ	0.132	**<0.001**	0.872	**<0.001**
T2	56.71 ± 41.16ᵇ	73.04 ± 35.85ᵃ	**0.002**			
T4	78.71 ± 33.88ᵃ	71.13 ± 36.35ᵃ	0.108			
ΔT2–T1 (95% CI)	−19.53 (−27.10 to −11.96)	3.79 (−1.09 to 8.68)				
ΔT4–T1 (95% CI)	2.47 (−3.61 to 8.54)	1.88 (−3.03 to 6.79)				
Within-group comparison (p**)	**<0.001**	**0.048**				
Vitality						
T1	53.15 ± 18.64ᵃ	55.32 ± 19.17ᵃ	0.356	**<0.001**	0.522	**0.004**
T2	49.05 ± 18.49ᵇ	53.12 ± 21.03ᵃ	0.091			
T4	55.69 ± 19.15ᵃ	53.33 ± 20.49ᵃ	0.340			
ΔT2–T1 (95% CI)	−4.10 (−7.21 to −0.99)	−2.20 (−5.06 to 0.67)				
ΔT4–T1 (95% CI)	2.54 (0.01 to 5.07)	−1.99 (−4.65 to 0.67)				
Within-group comparison (p**)	**0.001**	**0.024**				
Mental health						
T1	63.74 ± 18.56ᵃ	63.20 ± 16.79ᵃ	0.807	**0.006**	0.896	0.408
T2	60.52 ± 17.79ᵇ	61.99 ± 17.08ᵇ	0.482			
T4	63.56 ± 18.42ᵃ	63.39 ± 17.90ᵃ	0.940			
ΔT2–T1 (95% CI)	−3.22 (−5.60 to −0.84)	−1.21 (−3.20 to 0.77)				
ΔT4–T1 (95% CI)	−0.18 (−2.80 to 2.44)	0.19 (−1.88 to 2.26)				
Within-group comparison (p**)	**0.004**	**0.021**				
Social functioning						
T1	75.24 ± 22.68ᵃ	71.55 ± 22.51ᵃ	0.189	**<0.001**	0.425	**0.021**
T2	67.14 ± 23.78ᵇ	69.16 ± 23.31ᵃ	0.455			
T4	73.52 ± 23.11ᵃ	69.54 ± 22.94ᵃ	0.151			
ΔT2–T1 (95% CI)	−8.10 (−12.11 to −4.09)	−2.39 (−5.24 to 0.46)				
ΔT4–T1 (95% CI)	−1.72 (−5.46 to 2.02)	−2.02 (−5.34 to 1.31)				
Within-group comparison (p**)	**<0.001**	**0.002**				
Bodily pain						
T1	74.85 ± 23.00ᵃ	75.88 ± 20.74ᵃ	0.691	**0.039**	0.439	0.417
T2	71.39 ± 23.38ᵇ	74.75 ± 22.40ᵃ	0.187			
T4	74.67 ± 22.40ᵃ	75.79 ± 20.54ᵃ	0.662			
ΔT2–T1 (95% CI)	−3.45 (−6.73 to −0.18)	−1.12 (−3.77 to 1.53)				
ΔT4–T1 (95% CI)	−0.17 (−3.55 to 3.21)	−0.09 (−2.44 to 2.27)				
Within-group comparison (p**)	**0.041**	0.084				
General health perception						
T1	63.66 ± 17.30	62.46 ± 19.20	0.598	0.744	0.370	0.644
T2	64.11 ± 17.70	62.13 ± 19.39	0.378			
T4	64.73 ± 18.16	62.32 ± 19.29	0.317			
ΔT2–T1 (95% CI)	0.46 (−1.51 to 2.42)	−0.33 (−2.11 to 1.45)				
ΔT4–T1 (95% CI)	1.07 (−1.05 to 3.19)	−0.14 (−1.80 to 1.51)				
Within-group comparison (p**)	0.741	0.816				

Analyses were performed using two-way repeated measures ANOVA. Δ difference values represent the between-group difference in change from baseline (fasting minus non-fasting). Change scores (Δ) were calculated for each time interval (T2–T1, T3–T1, and T4–T1). Between-group comparisons of these change scores were performed using an independent samples *t*-test. In addition, repeated measures ANOVA was used to assess overall time, group, and time × group interaction effects. Values are presented as mean difference with 95% confidence intervals (CIs). A *p*-value <0.05 was considered statistically significant. T1: pre-Ramadan; T2: mid-Ramadan; T3: end of Ramadan; T4: 2 weeks after Ramadan, a,b,c,d Post-hoc Bonferrini. a: There is a significant difference with T1. b: There is a significant difference with T2. c: There is a significant difference with T3. d: There is a significant difference with T4. Covariates: Age and pre-Ramadan body weight. Bold values indicate statistically significant differences (*p* < 0.05).

Comparisons of change scores across groups (Table 5) show that the most pronounced differences emerged during the Ramadan period and were particularly concentrated in the areas of physical role, emotional role, and social functioning; however, these differences disappeared after Ramadan.

**Table 5:** Between-group differences in change scores.

Variable	Comparison	Δ difference (Fasting – Non-fasting)	*p*
Physical functioning	ΔT2–T1	−3.69 (−6.43 to −0.95)	**0.009**
	ΔT4–T1	−0.09 (−2.85 to 2.67)	0.950
Physical role functioning	ΔT2–T1	−28.44 (−36.91 to −19.97)	**<0.001**
	ΔT4–T1	1.79 (−4.26 to 7.84)	0.560
Emotional role functioning	ΔT2–T1	−23.32 (−32.29 to −14.34)	**<0.001**
	ΔT4–T1	0.59 (−7.19 to 8.36)	0.882
Vitality	ΔT2–T1	−1.91 (−6.11 to 2.30)	0.373
	ΔT4–T1	4.53 (0.87 to 8.19)	**0.015**
Mental health	ΔT2–T1	−2.01 (−5.10 to 1.08)	0.202
	ΔT4–T1	−0.38 (−3.70 to 2.95)	0.824
Social functioning	ΔT2–T1	−5.71 (−10.61 to −0.81)	**0.022**
	ΔT4–T1	0.30 (−4.68 to 5.28)	0.906
Bodily pain	ΔT2–T1	−2.33 (−6.52 to 1.86)	0.275
	ΔT4–T1	−0.09 (−4.19 to 4.01)	0.966
General health perception	ΔT2–T1	0.79 (−1.85 to 3.43)	0.558
	ΔT4–T1	1.22 (−1.46 to 3.90)	0.372

Δ difference values represent the between-group difference in change from baseline (fasting minus non-fasting). Change scores (Δ) were calculated for each time interval (T2–T1, T3–T1, and T4–T1), and between-group comparisons were performed using an independent samples *t*-test. These analyses complement the repeated-measures framework by quantifying the magnitude and direction of change over time. Values are expressed as mean difference with 95% confidence intervals (CIs). Negative values indicate a greater reduction in the fasting group, and positive values indicate a greater increase. A two-sided *p*-value <0.05 was considered statistically significant. Bold values indicate statistically significant differences (*p* < 0.05).

Changes in dietary intake are presented in Table 6. A significant time × group interaction was observed for total energy intake and the majority of macronutrients, indicating that dietary habits changed differently between the two groups.

**Table 6:** Comparison of groups by energy, macronutrients, and amount of water consumed.

Time point	Fasting (*n* = 141) Mean ± SD	Non-fasting (*n* = 141) Mean ± SD	p*	Time	Group	Time × Group
Energy (kcal)						
T1	1632.14 ± 705.03ᵇᶜ	1659.70 ± 651.93	0.732	**<0.001**	0.320	**<0.001**
T2	1468.75 ± 530.06ᵃᵈ	1626.41 ± 561.75	**0.015**			
T3	1456.25 ± 559.68ᵃᵈ	1623.46 ± 627.95	**0.020**			
T4	1715.01 ± 668.63ᵇᶜ	1608.16 ± 548.27	0.153			
ΔT2–T1 (95% CI)	−163.39 (−248.32 to −78.47)	−33.29 (−117.04 to 50.46)				
ΔT3–T1 (95% CI)	−175.89 (−267.54 to −84.25)	−36.24 (−150.60 to 78.12)				
ΔT4–T1 (95% CI)	82.87 (−11.09 to 176.83)	−51.54 (−149.90 to 46.82)				
Within-group comparison (p**)	**<0.001**	0.691				
Protein (g)						
T1	71.23 ± 34.61ᵇᶜ	74.52 ± 38.32	0.449	**<0.001**	**0.040**	**0.015**
T2	62.63 ± 25.94ᵃᵈ	73.01 ± 37.26	**0.008**			
T3	61.04 ± 23.80ᵃᵈ	71.64 ± 28.94	**0.001**			
T4	71.13 ± 29.98ᵇᶜ	73.24 ± 35.05	0.593			
ΔT2–T1 (95% CI)	−8.60 (−13.31 to −3.89)	−1.52 (−7.07 to 4.04)				
ΔT3–T1 (95% CI)	−10.18 (−15.25 to −5.11)	−2.88 (−7.99 to 2.23)				
ΔT4–T1 (95% CI)	−0.09 (−4.63 to 4.44)	−1.28 (−6.80 to 4.23)				
Within-group comparison (p**)	**<0.001**	0.743				
Protein (%)						
T1	17.98 ± 4.51	18.52 ± 5.03	0.337	0.223	0.052	0.286
T2	17.72 ± 6.10	18.90 ± 7.99	0.159			
T3	17.97 ± 9.41	19.21 ± 10.65	0.275			
T4	17.98 ± 7.48	20.70 ± 13.40	0.066			
ΔT2–T1 (95% CI)	−0.26 (−1.57 to 1.06)	0.38 (−1.01 to 1.78)				
ΔT3–T1 (95% CI)	−0.00 (−1.78 to 1.77)	0.69 (−1.20 to 2.58)				
ΔT4–T1 (95% CI)	0.00 (−1.39 to 1.39)	2.18 (−0.08 to 4.44)				
Within-group comparison (p**)	0.930	0.135				
Fats (g)						
T1	75.45 ± 40.51ᵇᶜ	77.98 ± 40.31	0.594	**0.029**	0.190	**0.007**
T2	64.54 ± 25.44ᵃᵈ	76.37 ± 31.01	**0.001**			
T3	69.66 ± 27.95ᵃᵈ	76.08 ± 48.50	0.159			
T4	79.09 ± 38.10ᵇᶜ	74.41 ± 30.06	0.261			
ΔT2–T1 (95% CI)	−10.91 (−16.91 to −4.91)	−1.61 (−8.00 to 4.78)				
ΔT3–T1 (95% CI)	−5.79 (−12.38 to 0.80)	−1.90 (−11.48 to 7.68)				
ΔT4–T1 (95% CI)	3.64 (−3.65 to 10.92)	−3.57 (−10.57 to 3.43)				
Within-group comparison (p**)	**<0.001**	0.207				
Fats (%)						
T1	39.87 ± 8.79ᶜ	40.57 ± 9.39	0.520	0.459	0.389	**0.037**
T2	38.98 ± 11.23	44.58 ± 42.36	0.147			
T3	41.71 ± 11.63ᵃ	39.20 ± 10.06	0.056			
T4	39.72 ± 8.75	40.11 ± 10.54	0.735			
ΔT2–T1 (95% CI)	−0.88 (−2.94 to 1.17)	4.00 (−3.13 to 11.14)				
ΔT3–T1 (95% CI)	1.84 (−0.22 to 3.91)	−1.37 (−3.32 to 0.57)				
ΔT4–T1 (95% CI)	−0.15 (−1.96 to 1.67)	−0.47 (−2.41 to 1.47)				
Within-group comparison (p**)	**0.046**	0.207				
Carbohydrates (g)						
T1	163.89 ± 76.16ᵃ	163.63 ± 68.75ᵃ	0.978	**<0.001**	0.960	**<0.001**
T2	155.35 ± 72.76ᵃ	158.56 ± 64.68ᵃ	0.690			
T3	146.29 ± 73.03ᵇ	163.05 ± 62.32ᵃ	**0.036**			
T4	179.68 ± 83.60ᶜ	161.37 ± 67.40ᵃ	**0.044**			
ΔT2–T1 (95% CI)	−8.54 (−19.66 to 2.58)	−5.07 (−14.71 to 4.57)				
ΔT3–T1 (95% CI)	−17.59 (−28.98 to −6.20)	−0.59 (−12.02 to 10.84)				
ΔT4–T1 (95% CI)	15.79 (5.12 to 26.46)	−2.26 (−12.69 to 8.16)				
Within-group comparison (p**)	**<0.001**	0.774				
Carbohydrates (%)						
T1	40.64 ± 8.51	39.81 ± 9.49	0.432	0.115	0.536	0.538
T2	44.51 ± 19.76	41.72 ± 25.16	0.285			
T3	42.92 ± 27.61	44.02 ± 29.09	0.748			
T4	44.47 ± 23.77	42.03 ± 27.38	0.401			
ΔT2–T1 (95% CI)	3.87 (0.38 to 7.35)	1.92 (−2.53 to 6.36)				
ΔT3–T1 (95% CI)	2.28 (−2.48 to 7.04)	4.22 (−0.95 to 9.38)				
ΔT4–T1 (95% CI)	3.83 (−0.31 to 7.96)	2.22 (−2.55 to 6.98)				
Within-group comparison (p**)	0.114	0.359				
Fiber (g)						
T1	20.72 ± 10.50ᵇᶜ	19.93 ± 10.11	0.520	**<0.001**	0.534	**0.002**
T2	17.20 ± 8.44ᵃᵈ	19.15 ± 8.87	0.067			
T3	16.94 ± 8.45ᵃᵈ	19.14 ± 8.67	**0.041**			
T4	21.00 ± 9.91ᵇᶜ	19.83 ± 10.03	0.341			
ΔT2–T1 (95% CI)	−3.52 (−5.32 to −1.71)	−0.77 (−2.29 to 0.74)				
ΔT3–T1 (95% CI)	−3.77 (−5.32 to −2.23)	−0.78 (−2.55 to 0.98)				
ΔT4–T1 (95% CI)	0.28 (−1.12 to 1.69)	−0.10 (−1.94 to 1.74)				
Within-group comparison (p**)	**<0.001**	0.629				
Water (ml)						
T1	1959.36 ± 670.57ᵇᶜ	1897.52 ± 653.43ᵈ	0.437	**<0.001**	0.218	**<0.001**
T2	1693.76 ± 601.74ᵃᵈ	1928.01 ± 660.12	**0.002**			
T3	1799.72 ± 613.42ᵃᵈ	1933.69 ± 661.75	0.085			
T4	1960.35 ± 639.13ᵇᶜ	1996.45 ± 675.80ᵃ	0.653			
ΔT2–T1 (95% CI)	−265.60 (−353.08 to −178.13)	30.50 (−37.80 to 98.80)				
ΔT3–T1 (95% CI)	−159.65 (−250.16 to −69.13)	36.17 (−37.28 to 109.62)				
ΔT4–T1 (95% CI)	0.99 (−66.00 to 67.99)	98.94 (28.04 to 169.83)				
Within-group comparison (p**)	**<0.001**	**0.035**				

Analyses were performed using two-way repeated measures ANOVA. Δ difference values represent the between-group difference in change from baseline (fasting minus non-fasting). Change scores (Δ) were calculated for each time interval (T2–T1, T3–T1, and T4–T1). Between-group comparisons of these change scores were performed using an independent samples *t*-test. In addition, repeated measures ANOVA was used to assess overall time, group, and time × group interaction effects. Values are presented as mean difference with 95% confidence intervals (CIs). A *p*-value <0.05 was considered statistically significant. T1: pre-Ramadan; T2: mid-Ramadan; T3: end of Ramadan, T4: 2 weeks after Ramadan. a,b,c,d Post-hoc Bonferrini. a: There is a significant difference with T1. b: There is a significant difference with T2. c: There is a significant difference with T3. d: There is a significant difference with T4. Covariates: Age and pre-Ramadan body weight. Bold values indicate statistically significant differences (*p* < 0.05).

Energy intake in the fasting group decreased significantly during Ramadan (ΔT2–T1: −163.39 kcal; ΔT3–T1: −175.89 kcal) and increased again after Ramadan. In contrast, energy intake in the non-fasting group remained relatively stable. Comparisons between the groups reveal that the reduction in energy intake was more pronounced in the fasting group during the Ramadan period. Intake of protein, fat, and fiber also decreased in the fasting group during Ramadan, but no significant change was observed in the non-fasting group. Water consumption is one of the most striking differences between the groups. Water intake in the fasting group decreased significantly during Ramadan (ΔT2–T1: −265.6 mL), whereas a slight increase was observed in the non-fasting group. This difference is statistically significant. As for the percentage distribution of macronutrients, no significant change was observed over time in either group. This suggests that the Ramadan fast has an effect on total intake rather than on the composition of the diet. The temporal changes in energy and nutrient intake are also illustrated in Supplementary Figure S2.

Finally, comparisons of dietary change scores between groups (Table 7) confirm that the observed differences were largely due to reductions in energy, macronutrient, fiber, and water intake in the fasting group during Ramadan, and that these effects largely disappeared after Ramadan.

**Table 7:** Between-group differences in change scores.

Variable	Comparison	Δ difference (Fasting – Non-fasting)	p
Energy (kcal)	ΔT2–T1	−130.10 (−248.86 to −11.34)	**0.032**
	ΔT3–T1	−139.66 (−285.60 to 6.29)	0.061
	ΔT4–T1	134.41 (−1.03 to 269.85)	0.052
Protein (g)	ΔT2–T1	−7.08 (−14.34 to 0.17)	0.056
	ΔT3–T1	−7.30 (−14.47 to −0.13)	**0.046**
	ΔT4–T1	1.19 (−5.92 to 8.30)	0.742
Protein (%)	ΔT2–T1	−0.64 (−2.55 to 1.27)	0.510
	ΔT3–T1	−0.69 (−3.28 to 1.89)	0.597
	ΔT4–T1	−2.18 (−4.82 to 0.47)	0.106
Fats (g)	ΔT2–T1	−9.30 (−18.03 to −0.57)	**0.037**
	ΔT3–T1	−3.89 (−15.47 to 7.70)	0.509
	ΔT4–T1	7.20 (−2.86 to 17.27)	0.160
Fats (%)	ΔT2–T1	−4.89 (−12.30 to 2.53)	0.195
	ΔT3–T1	3.22 (0.39 to 6.05)	**0.026**
	ΔT4–T1	0.32 (−2.32 to 2.96)	0.812
Carbohydrates (g)	ΔT2–T1	−3.47 (−18.13 to 11.19)	0.642
	ΔT3–T1	−17.01 (−33.07 to −0.94)	**0.038**
	ΔT4–T1	18.06 (3.21 to 32.90)	**0.017**
Carbohydrates (%)	ΔT2–T1	1.95 (−3.67 to 7.57)	0.495
	ΔT3–T1	−1.94 (−8.93 to 5.06)	0.586
	ΔT4–T1	1.61 (−4.68 to 7.89)	0.615
Fiber (g)	ΔT2–T1	−2.74 (−5.09 to −0.40)	**0.022**
	ΔT3–T1	−2.99 (−5.33 to −0.66)	**0.012**
	ΔT4–T1	0.38 (−1.92 to 2.68)	0.746
Water (ml)	ΔT2–T1	−296.10 (−406.63 to −185.57)	**<0.001**
	ΔT3–T1	−195.82 (−311.90 to −79.74)	**0.001**
	ΔT4–T1	−97.94 (−195.06 to −0.82)	**0.048**

Δ difference values represent the between-group difference in change from baseline (fasting minus non-fasting). Change scores (Δ) were calculated for each time interval (T2–T1, T3–T1, and T4–T1), and between-group comparisons were performed using an independent samples *t*-test. These analyses complement the repeated-measures framework by quantifying the magnitude and direction of change over time. Values are expressed as mean difference with 95% confidence intervals (CIs). Negative values indicate a greater reduction in the fasting group, and positive values indicate a greater increase. A two-sided *p*-value <0.05 was considered statistically significant. Bold values indicate statistically significant differences (*p* < 0.05).

## 4 Discussion

The current study compared individuals who fasted during Ramadan with those who did not in terms of body weight, daytime sleepiness, physical activity, quality of life, and food intake across four time points. The findings suggest that fasting status was associated with differences, particularly in physical activity, daytime sleepiness, and certain quality-of-life dimensions during the Ramadan period. Energy and macronutrient intakes as well as body weight showed temporary reductions during Ramadan and tended to return toward pre-Ramadan levels afterwards. Similarly, several SF-36 subdimensions, particularly physical functioning and physical role functioning, declined during mid-Ramadan and recovered after Ramadan. These findings indicate that lifestyle changes during the fasting month, including alterations in daytime sleepiness, meal timing, and daily routines, may lead to temporary fluctuations in perceived physical functioning. Overall, although several short-term changes were observed during Ramadan, most outcomes were largely similar between fasting and non-fasting individuals 2 weeks after Ramadan. When change-from-baseline (*Δ*) values and 95% confidence intervals were considered, the temporary nature and trajectory of these changes became more apparent, with most outcomes showing deterioration during Ramadan and recovery after Ramadan. A characteristic pattern was observed, particularly in the fasting group, where several parameters showed a decline during Ramadan, followed by a rebound toward baseline levels after Ramadan.

This study found that changes in body weight over time differed between individuals who fasted and those who did not. Although the fasting group had a higher body weight at the beginning, the difference between the two groups disappeared during and after Ramadan. This suggests that Ramadan fasting may have a temporary regulatory effect on body weight. Intra-group analyses also showed that body weight changed significantly over time only in the fasting group. In particular, the *Δ* analyses showed that weight reduction was more pronounced during mid- and late-Ramadan in the fasting group, whereas body weight remained relatively stable in the non-fasting group. These findings are consistent with studies in the literature. Urooj and colleagues reported that body weight decreased at the end of Ramadan compared to before Ramadan (17). Similarly, Hajek and colleagues showed that there was an average weight loss of approximately 1 kg during Ramadan, but this loss was regained in a short time (18). Other studies also reported that body weight returned to pre-Ramadan levels within 2–5 weeks after Ramadan (19, 20). When these results are considered together, it can be said that Ramadan fasting may cause temporary decreases in body weight, but this effect disappears when individuals return to their normal eating and lifestyle habits after Ramadan. Therefore, long-term dietary and lifestyle changes are important for sustainable weight control.

Fasting can affect an individual’s circadian rhythm. Restricting food and drink intake during fasting may disrupt the sleep–wake cycle (21). At the same time, consuming iftar and suhoor meals late at night may negatively affect daytime sleepiness (22). In the study by Faris et al., a decrease of approximately 1 h in total sleep time and an increase of approximately 1 point in ESS were observed during Ramadan (23). In the study by Bahammam et al., no significant change in scale scores was observed. However, the percentage of rapid eye movement (REM) sleep was reported to decrease significantly during fasting (21). Similarly, Trabelsi et al. reported that the percentage of participants experiencing excessive daytime sleepiness increased from 3.8% before Ramadan to 7.7% during Ramadan, and total sleep time decreased by approximately 1 h (24). The present study found that the scale scores of fasting participants were significantly higher than those of non-fasting participants at the middle and end of Ramadan (*p* = 0.006 and *p* = 0.010, respectively). In addition, there was an increase in the scale scores of those who fasted during Ramadan compared to the pre-Ramadan period. A decrease was observed 2 weeks after Ramadan (*p* < 0.0125). The change-from-baseline analyses also showed that the increase in daytime sleepiness was more marked in fasting individuals during Ramadan and tended to return toward baseline after Ramadan. These results suggest that the deterioration in daytime sleepiness during Ramadan is due to the biological changes caused by fasting and is temporary. Necessary measures should be taken to improve daytime sleepiness during this period. Taking care to go to bed and wake up at similar times each day may ensure that the body clock is less affected. Short daytime sleep (20–30 min) can also increase energy levels. In addition, the suppression of melatonin release can be prevented by reducing the use of phones, tablets, and computers that emit blue light after iftar.

In addition to changes in eating and drinking habits, Ramadan fasting can affect circadian rhythms (sleep/wake cycles), metabolism, and hormone secretion. These changes, together with insomnia, can also affect physical performance (25). A study showing that Ramadan fasting affects the daily physical activity of Muslims reported that the average number of steps per day of fasting participants was significantly reduced (26). In another study conducted by Geok et al., it was observed that the level of physical activity was significantly reduced during Ramadan, resulting in a significant difference when compared to the pre- and post-feasting periods (27). Similarly, in the present study, the total physical activity score of fasting individuals was found to be significantly lower than that of the non-fasting group in the middle (*p* = 0.010) and at the end (*p* = 0.048) of Ramadan. Among those who fasted, the physical activity score decreased during Ramadan compared to the pre-Ramadan period (*p* < 0.0125). Two weeks after Ramadan, the physical activity score was similar to that before Ramadan. Similarly, PAL-based change scores indicated a clearer reduction during Ramadan in the fasting group, followed by recovery after Ramadan, whereas the non-fasting group showed only limited fluctuation. It can be said that strategies to support physical activity during this period should be developed, especially considering daytime sleepiness and metabolic changes. To maintain physical activity during Ramadan, low-to-moderate intensity walking or light resistance exercises can be performed 1–2 h after iftar.

In the literature, a study conducted on non-fasting individuals with type 1 diabetes reported that sleep duration decreased and physical activity levels declined during Ramadan (28). In the present study, significant changes over time were observed in ESS scores (*p* = 0.004) and total physical activity levels (*p* = 0.032) in the non-fasting group. These findings suggest that family and social environment may indirectly influence the sleep and physical activity habits of non-fasting individuals in accordance with the Ramadan routine. Therefore, measures to support sleep and physical activity during Ramadan are important not only for fasting individuals but also for non-fasting individuals who are exposed to Ramadan routines through their social environment.

Quality of life may fluctuate over time depending on changes in daily routines, lifestyle habits, and psychosocial factors. In the present study, a significant time effect was observed in several SF-36 subdimensions, including physical functioning, physical role functioning, emotional role functioning, vitality, mental health, and social functioning. Overall, quality-of-life scores tended to decrease during mid-Ramadan and returned to levels similar to the pre-Ramadan period 2 weeks after Ramadan. This finding suggests that certain aspects of quality of life may temporarily decline during the Ramadan fasting period but tend to recover after the fasting month. Similar temporal fluctuations during Ramadan have also been reported in previous studies investigating lifestyle changes during the fasting month (29, 30).

The *Δ* analyses further showed that the most pronounced deteriorations were observed during Ramadan and were largely attenuated after Ramadan, supporting the transient nature of these quality-of-life changes. However, no overall group effect was observed for any SF-36 subdimension, indicating that the general quality-of-life levels of fasting and non-fasting individuals were comparable. Nevertheless, significant time × group interactions in several domains suggest that the pattern of change across the Ramadan period differed between the two groups. In particular, fasting individuals showed a more pronounced decline in some functional and psychosocial domains during mid-Ramadan, followed by a return to baseline levels after Ramadan. These temporary changes may be related to alterations in daytime sleepiness, circadian rhythm, and meal timing commonly observed during Ramadan (21, 31).

Previous studies examining the effect of Ramadan fasting on quality of life have reported inconsistent findings. For example, Khalili et al. reported significant increases in several SF-36 subdimensions during Ramadan compared to the pre-Ramadan period. In contrast, the present study observed a temporary decrease in certain quality-of-life domains during mid-Ramadan. Differences between studies may be explained by variations in study populations, cultural context, physical activity levels, and individual attitudes toward fasting. Ramadan is often associated with positive spiritual experiences as well as changes in daily routines, including daytime sleepiness and eating habits. The balance between these factors may influence individuals’ perceived quality of life (9, 32).

In the present study, bodily pain showed only a significant time effect, while group and time × group interaction effects were not significant. This finding suggests that Ramadan fasting may not have a substantial impact on pain perception. Previous studies have also reported limited effects of Ramadan fasting on inflammatory markers and physical symptoms (32). Similarly, general health perception remained stable throughout the study period, indicating that although some functional aspects of quality of life may fluctuate during Ramadan, individuals’ overall perception of their health appears to remain relatively unchanged. These findings suggest that the impact of Ramadan fasting on certain dimensions of quality of life may be transient and largely related to short-term lifestyle adjustments during the fasting period. Therefore, maintaining healthy daytime sleepiness, balanced nutrition, and regular physical activity after Ramadan may help sustain potential health benefits.

This study found that changes in food intake are not only time-dependent but also follow a different pattern depending on fasting status. The time × group interaction observed in energy and some macronutrient intakes in the analyses indicates that the Ramadan period affected dietary behaviors, particularly in individuals who fasted. Indeed, a decrease in energy, macronutrients (g/day), fiber, and water intake was observed in fasting individuals during Ramadan, while no similar change was observed in the non-fasting group. This suggests that changes in food intake, particularly in fasting individuals, are due to the reduction in the number of meals and the restriction of food consumption to specific hours during Ramadan. Furthermore, the disappearance of differences between the groups after Ramadan indicates that these changes are temporary and that nutrient intake may return to previous levels as daily eating patterns return to normal. In line with the results of our study, studies conducted in Turkey have reported a decrease in total calorie, carbohydrate, and protein intake during Ramadan (33, 34). In contrast, some studies have reported an increase in total calorie and protein intake during Ramadan (35–37). A meta-analysis of studies conducted in Western and Eastern Asian populations reported that daily energy intake mostly decreased during Ramadan, but daily energy intake significantly increased in African populations (20). These differences may stem from the geographical, seasonal, and cultural characteristics of the studies. Furthermore, differences in the methods used to assess nutrient intake may also contribute to the conflicting results in the literature. This interpretation was also supported by the change-from-baseline analyses, which showed that the largest between-group differences emerged during Ramadan and were substantially reduced after Ramadan.

Strengths of the study: One of the key strengths of this study is the inclusion of a non-fasting comparison group, which allowed us to distinguish changes related to fasting from those associated with the broader Ramadan environment. Previous studies have mostly focused on a single parameter, such as anthropometric measurements or psychological effects of dietary changes during Ramadan. In the present study, the physical and psychological effects were analyzed separately through subdimensions, which ensured that the results were clear and detailed. The study provides a holistic perspective by evaluating quality of life, sleep, physical activity, and nutritional status together.

Limitations of the study: Several limitations should be considered when interpreting the findings. First, the fasting and non-fasting groups differed in some baseline lifestyle-related characteristics, including alcohol consumption, smoking status, and marital status. These differences may reflect broader behavioral and psychosocial differences between the groups and may have confounded the observed associations. Therefore, the between-group differences cannot be attributed solely to fasting status. Second, dietary intake was self-reported, and the study relied largely on subjective assessments, which may have increased the risk of reporting error, recall bias, and social desirability bias. Furthermore, objective assessments such as actigraphy and biomarkers were not included. Another limitation is that Ramadan-specific religious practices, such as tarawih prayers, were not specifically assessed, although these practices may contribute to daily physical activity patterns. In addition, participants’ religious affiliations were not specifically recorded; therefore, the potential impact of religious background on lifestyle behaviors could not be evaluated. As this study was observational and non-randomized, causal relationships between Ramadan fasting and the observed outcomes cannot be established. Finally, because the study was conducted in Istanbul and involved relatively young and healthy adults, the findings may not be generalizable to other cultural or geographic settings, other seasonal contexts, older adults, or individuals with chronic diseases.

## 5 Conclusion

This study showed that Ramadan fasting was associated with temporary changes in daytime sleepiness, physical activity, dietary intake, and certain quality-of-life dimensions. Fasting individuals experienced higher daytime sleepiness and lower physical activity levels during Ramadan compared to non-fasting individuals. Energy and macronutrient intake, as well as body weight, decreased during the fasting period but tended to return toward pre-Ramadan levels afterwards. Similarly, some quality-of-life dimensions declined during mid-Ramadan and recovered after the fasting month. Overall, these findings suggest that most lifestyle changes observed during Ramadan are short term and reversible. Further studies with larger samples and objective measurement methods are needed to better understand the long-term health implications of Ramadan fasting.

## Data Availability

The data supporting the findings of this study are available from the corresponding author upon reasonable request.
