# Supplementary material for: Nutritional, sleep, physical activity, and quality-of-life changes during Ramadan fasting: a prospective comparative study
Source: Front Nutr. 2026 May 4;13:1809040. doi: 10.3389/fnut.2026.1809040 (PMC13180933; doi:10.3389/fnut.2026.1809040)
Supplement: Supplementary file 1 [file Data_Sheet_1.PDF]

EK 3- EPWORTH UYKU ÖLÇEĞİ

Tarih:

EPWORTH UYKU ÖLÇEĞİ

**DURUM**	**0 (Hiçbir zaman uyuklamam)**	**1 (Nadiren Uyuklarım)**	**2 (Zaman zaman uyuklarım)**	**3 (Büyük olasılıkla uyuklarım)**
Oturmuş bir şeyler okurken				
Televizyon seyrederken				
Toplum içinde hareketsizce otururken. (örneğin: herhangi bir toplantıda veya tiyatro gibi yerlerde)				
Ara vermeden en az bir saat süren bir araba yolculuğunda yolcu olarak bulunurken				
Öğleden sonra koşullar uygun olduğunda, dinlenmek için uzanmışken				
Birisiyle oturmuş konuşurken				
Alkol almadığım bir öğle yemeğinden sonra sessizce otururken				
İçinde olduğum araba, trafikte birkaç dakika için durduğunda				
**TOPLAM**				

DEĞERLENDİRME;

<10 = NORMAL

10-15= UYKULULUK HALİ
