# Supplementary material for: Nutritional, sleep, physical activity, and quality-of-life changes during Ramadan fasting: a prospective comparative study
Source: Front Nutr. 2026 May 4;13:1809040. doi: 10.3389/fnut.2026.1809040 (PMC13180933; doi:10.3389/fnut.2026.1809040)
Supplement: Supplementary file 2 [file Data_Sheet_2.PDF]

EK 4- BESİN TÜKETİM KAYDI ***Tarih:***

**ÖĞÜN**	**MİKTAR / ÖLÇÜ**	**BESİNLER**
Örnek:<br><br>***AKŞAM***<br>***19.00***	***6 yemek kaşığı /1 çukur tabak***<br>***3 yemek kaşığı***<br>***4 yemek kaşığı***<br>***1 küçük kase (1 tatlı kaşığı zeytinyağı)***	***Sulu etli bezelye***<br>***Pirinç pilavı,***<br>***Tam yağlı yoğurt***<br>***Zeytinyağlı salata***
**Uyanınca**		
**Kahvaltı**		
**Ara**		
**Öğle yemeği**		
**Ara**		
**Akşam yemeği**		
**Ara**		

Günlük su tüketimi:

RAMAZAN AYINDA BESİN TÜKETİM KAYDI (ORUÇ TUTAN GRUP İÇİN)

ÖĞÜN	***MİKTAR / ÖLÇÜ***	***BESİNLER***
İftar		
Ara		
Sahur		

Günlük su tüketimi:
