# Supplementary material for: Nutritional, sleep, physical activity, and quality-of-life changes during Ramadan fasting: a prospective comparative study
Source: Front Nutr. 2026 May 4;13:1809040. doi: 10.3389/fnut.2026.1809040 (PMC13180933; doi:10.3389/fnut.2026.1809040)
Supplement: Supplementary file 3 [file Data_Sheet_3.PDF]

EK 5

Tarih:

24 SAATLİK FİZİKSEL AKTİVİTE KAYIT FORMU

AKTİVİTE TÜRÜ	SÜRE			
	**Aktivite Katsayısı (PAR)**	**Saat**	**Dakika**	**Toplam REE faktörü**
Dinlenme (Uyku, uzanma)	1.0			
Çok hafif aktivite (Oturarak çalışma; boya yapma, araba kullanma, laboratuvar, dikiş, örgü, ütü, yemek yapma, masa başı oyun, müzik aleti çalma, tv seyretme)	1.5			
Hafif aktivite (yavaş yürüme, marangoz işleri, lokanta işleri, ev temizliği, çocuk bakımı, yelken, masa tenisi vb.)	2.5			
Orta aktiviteler (hızlı yürüme, tarla işleri, yük taşıma, bisiklete binme, kayak, tenis, dans)	5.0			
Ağır aktivite (yokuş yukarı yük taşıma, elle yorucu kazma işi, basketbol, tırmanma, futbol, inşaat işçiliği)	7.0			
**TOPLAM**		**24**	**1440**	
