# Supplementary material for: Nutritional, sleep, physical activity, and quality-of-life changes during Ramadan fasting: a prospective comparative study
Source: Front Nutr. 2026 May 4;13:1809040. doi: 10.3389/fnut.2026.1809040 (PMC13180933; doi:10.3389/fnut.2026.1809040)
Supplement: Supplementary file 4 [file Data_Sheet_4.PDF]

EK 6

Tarih:

YAŞAM KALİTESİ ÖLÇEĞİ SF-36

Aşağıdaki sorular sizin kendi sağlığınız hakkındaki görüşünüzü, kendinizi nasıl hissettiğinizi ve günlük aktivitelerinizi ne kadar yerine getirebildiğinizi öğrenmek amacındadır.

1. Genel olarak sağlığınız için aşağıdakilerden hangisini söyleyebilirsiniz?

Mükemmel (1) Çok iyi (2) İyi (3) Orta (4) Kötü (5)

2. Bir yıl öncesi ile karşılaştırdığınızda şu anki genel sağlık durumunuzu nasıl değerlendirirsiniz?

Çok daha iyi (1) Biraz iyi (2) Hemen hemen aynı (3)

Biraz daha kötü (4) Çok daha kötü (5)

Aşağıdaki sorular bir gün içinde yapabileceğiniz işlerle (aktivitelerle) ilgilidir. Sağlığınız bu aktiviteleri kısıtlıyor mu? Eğer kısıtlıyorsa, ne kadar?

	**Evet, çok kısıtlı**	**Evet, biraz kısıtlı**	**Hayır, hiç kısıtlı değil**
3. Koşmak, ağır kaldırmak, ağır sporlara katılmak gibi ağır etkinlikler	1	2	3
4. Bir masayı çekmek, elektrik süpürgesini itmek ve ağır olmayan sporları yapmak gibi orta dereceli etkinlikler	1	2	3
5. Market poşetlerini kaldırmak veya taşımak	1	2	3
6. Birkaç kat merdiven çıkmak	1	2	3
7. Bir kat merdiven çıkmak	1	2	3
8. Eğilmek, diz çökmek, çömelmek, diz çökmek	1	2	3
9. Bir kilometreden fazla yürümek	1	2	3
10. Birkaç yüz metre yürümek	1	2	3
11. Yüz metre yürümek	1	2	3
12. Kendi başına banyo yapmak ve giyinmek	1	2	3

Son 4 hafta boyunca bedensel sağlığınızın sonucu olarak, işiniz veya diğer günlük etkinliklerinizde, aşağıdaki sorunlardan biriyle karşılaştınız mı?

	**Evet**	**Hayır**
13. Çalışma yaşamınızda veya diğer aktivitelerinizde geçirdiğiniz zamanı kısalttınız mı?	1	2
14. Arzu ettiğinizden daha az şeyi mi tamamlayabildiniz?	1	2
15. Çalışma veya diğer yaptığınız işlerin çeşidinde kısıtlama yaptınız mı?	1	2
16. Çalışma yaşamınızda veya diğer aktivitelerinizi yapmakta güçlük çektiniz mi? (Aşırı efor -çaba sarf ettiniz mi?)	1	2

Son 4 hafta boyunca, duygusal sorunlarınızın (örneğin çökkünlük veya kaygı) sonucu olarak işiniz veya diğer günlük etkinliklerinizle ilgili aşağıdaki sorunlarla karşılaştınız mı?

	**Evet**	**Hayır**
17. Çalışma yaşamınızda veya diğer aktivitelerinizde geçirdiğiniz zamanı kısalttınız mı?	1	2
18. Arzu ettiğinizden daha az işi mi tamamlayabildiniz?	1	2
19. İşinizle veya diğer aktivitelerinizle ilgili işleri her zamanki kadar dikkat vererek yapamadınız mı?	1	2

20. Son 4 hafta boyunca bedensel sağlığınız veya duygusal sorunlarınız, aileniz, arkadaş veya komşularınızla olan olağan sosyal etkinliklerinizi ne kadar etkiledi?

Hiç etkilemedi (1) Çok Az (2) Orta Derecede (3) Epeyce (4) Çok fazla (5)

21. Son 4 hafta içinde vücudunuzda ne kadar ağrı oldu?

Hiç olmadı (1) Çok Az (2) Hafif (3) Orta (4) Çok (5) Pek çok (6)

22. Son 4 hafta boyunca ağrınız, normal işinizi (hem ev işlerinizi hem ev dışı işinizi düşününüz) ne kadar etkiledi?

Hiç Etkilemedi (1) Biraz Etkiledi (2) Orta Derecede (3)

Epey Etkiledi (4)   Çok Etkiledi (5)

Aşağıdaki sorular sizin son 4 hafta boyunca neler hissettiğinizle ilgilidir. Her soru için, sizin duygularınızı en iyi karşılayan yanıtı, son 4 haftadaki sıklığını göz önüne alarak seçiniz.

	**Sürekli**	**Çoğu zaman**	**Epey zaman**	**Bazen**	**Ara sıra**	**Hiçbir zaman**
23. Kendinizi yaşam dolu olarak hissettiniz mi?	1	2	3	4	5	6
24. Çok sinirli biri oldunuz mu?	1	2	3	4	5	6
25. Hiçbir şeyin size neşelendiremeyeceği kadar moraliniz bozuk ve kötü oldu mu?	1	2	3	4	5	6
26. Kendinizi sakin ve huzurlu hissettiniz mi?	1	2	3	4	5	6
27. Çok enerjik oldunuz mu?	1	2	3	4	5	6
28. Kendinizi kalbi kırık ve üzgün hissettiniz mi?	1	2	3	4	5	6
29. Kendinizi yıpranmış, bitkin hissettiniz mi?	1	2	3	4	5	6
30. Mutlu, sevinçli bir insan oldunuz mu?	1	2	3	4	5	6
31. Yorgunluk hissettiniz mi?	1	2	3	4	5	6

32. Son 4 hafta boyunca bedensel sağlığınız veya duygusal sorunlarınız sosyal etkinliklerinizi (arkadaş veya akrabalarınızı ziyaret etmek gibi) ne sıklıkta etkiledi?

Sürekli (1)   Çoğu zaman (2)   Bazen (3)   Ara Sıra (4)   Hiçbir zaman (5)

Aşağıdaki her bir ifade sizin için ne kadar doğru veya yanlıştır? Her bir ifade için en uygun olanını işaretleyiniz.

	**Kesinlikle doğru**	**Çoğunlukla doğru**	**Emin değilim**	**Çoğunlukla yanlış**	**Kesinlikle yanlış**
33. Ben diğer insanlara göre daha kolay hastalanıyorum.	1	2	3	4	5
34. Tanıdığım kişiler kadar sağlıklıyım	1	2	3	4	5
35. Sağlığımın kötüleşmekte olduğunu sanıyorum.	1	2	3	4	5
36. Sağlığım mükemmeldir.	1	2	3	4	5

SF 36 HESAPLAMA

**1-2-20-22-34-36. sorular için;*

1 = 100 puan

2 = 75 puan

3 =50 puan

4 = 25 puan

5 = 0 puan

**3-4-5-6-7-8-9-10-11-12.sorular için;*

1 = 0 puan

2 = 50 puan

3 =100 puan

**13-14-15-16-17-18-19.sorular için;*

1 = 0 puan

2 =100 puan

**21-23-26-27-30.sorular için;*

1 = 100 puan

2 = 80 puan

3 =60 puan

4 = 40 puan

5 = 20 puan

6 = 0 puan

**24-25-28-29-31.sorular için;*

1 = 0 puan

2 = 20 puan

3 = 40 puan

4 = 60 puan

5 = 80 puan

6 =100 puan

<u>32-33-35.sorular için;</u>

1= 0 puan

2 =25 puan

3 =50 puan

4= 75 puan

5= 100 puan

ALT PARAMETRELERE AİT DEĞERLERİ BULMAK İÇİN FORMÜL

**FİZİKSEL FONKSİYON**	(3+4+5+6+7+8+9+10+11+12) /10
**FİZİKSEL ROL GÜÇLÜĞÜ**	(13+14+15+16) /4
**EMOSYONEL ROL GÜÇLÜĞÜ**	(17 +18+19) /3
**ENERJİ/CANLILIK/VİTALİTE**	(23+27+29+31) /4
**RUHSAL SAĞLIK**	(24+25+26+28+30) /5
**SOSYAL İŞLEVSELLİK**	(20+32) /2
**AĞRI**	(21+22) /2
**GENEL SAĞLIK ALGISI**	(1+33+34+35+36) /5
