# Supplementary material for: Nutritional, sleep, physical activity, and quality-of-life changes during Ramadan fasting: a prospective comparative study
Source: Front Nutr. 2026 May 4;13:1809040. doi: 10.3389/fnut.2026.1809040 (PMC13180933; doi:10.3389/fnut.2026.1809040)
Supplement: Supplementary file 5 [file Supplementary_file_1.DOCX]

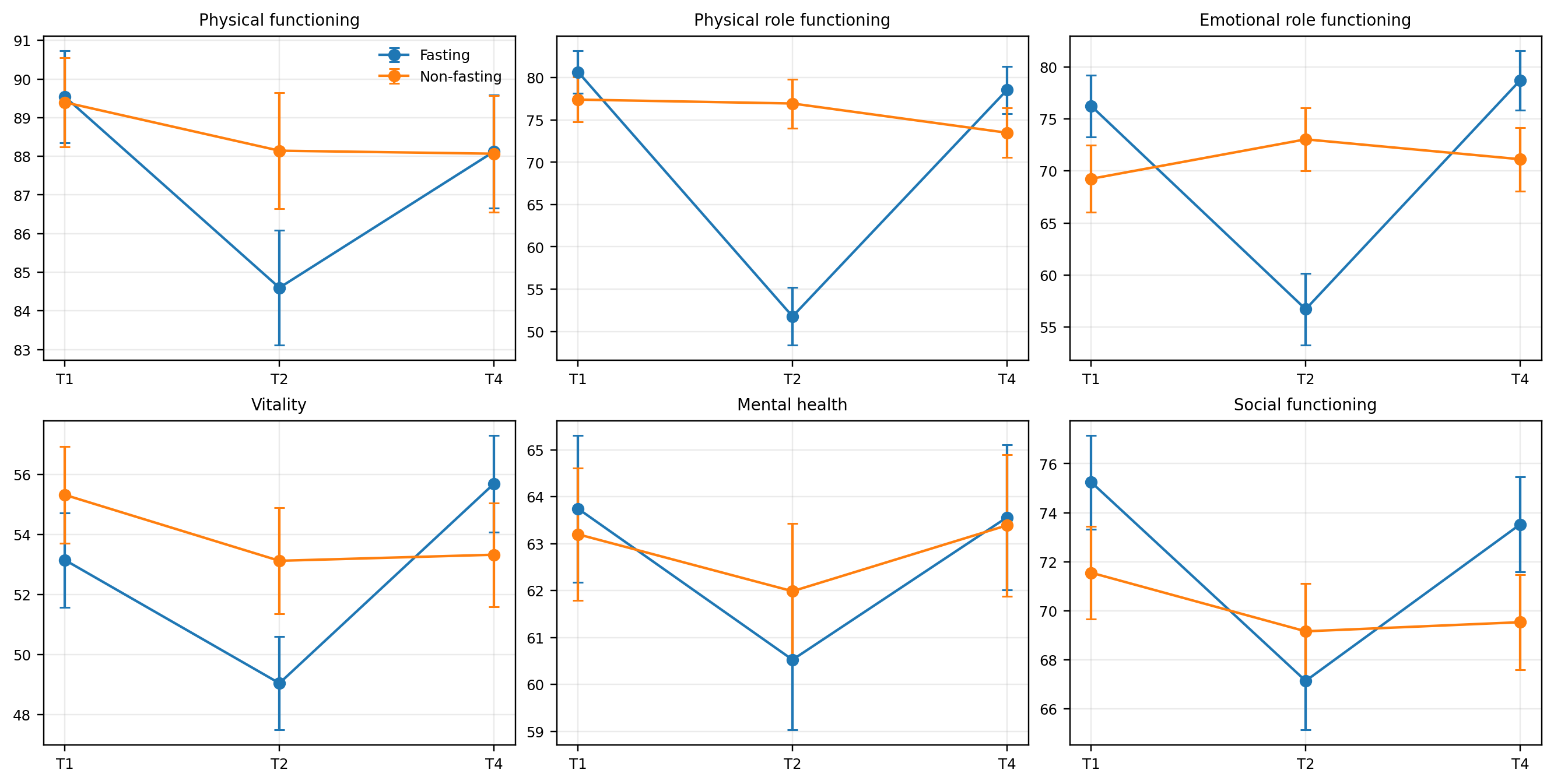


**Supplementary Figure S1.** Temporal changes in SF-36 subdimensions (physical functioning, physical role functioning, emotional role functioning, vitality, mental health, and social functioning) in fasting and non-fasting individuals across study time points (T1: pre-Ramadan, T2: mid-Ramadan, T3: end-Ramadan, T4: two weeks post-Ramadan).
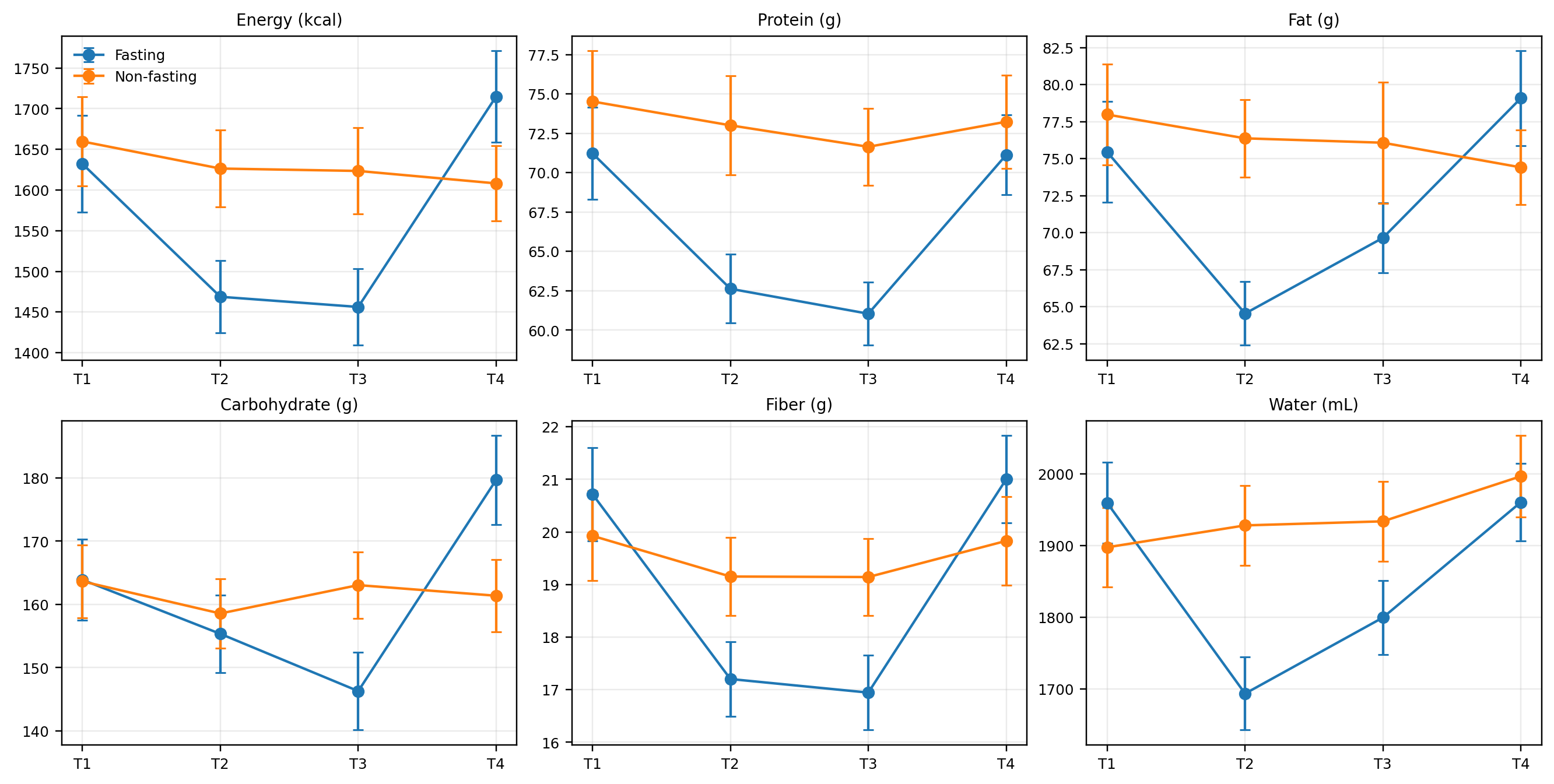


**Supplementary Figure S2.** Temporal changes in energy, macronutrient intake, fiber, and water consumption in fasting and non-fasting individuals across study time points.
